# Effects and Reversibility of Pre- and Post-natal Iron and Omega-3 Fatty Acid Deficiency, Alone and in Combination, on Bone Development in Rats

**DOI:** 10.3389/fnut.2021.802609

**Published:** 2022-01-17

**Authors:** Estelle Strydom, Lizelle Zandberg, Erna T. Kemp, Philip vZ. Venter, Cornelius M. Smuts, Herculina S. Kruger, Jeannine Baumgartner

**Affiliations:** ^1^Centre of Excellence for Nutrition, North-West University, Potchefstroom, South Africa; ^2^School of Mechanical and Nuclear Engineering, North-West University, Potchefstroom, South Africa; ^3^Laboratory of Human Nutrition, Institute of Food, Nutrition and Health, ETH Zürich, Zürich, Switzerland

**Keywords:** bone development, bone mineral density, bone strength, iron, omega-3 polyunsaturated fatty acid (n-3 PUFA)

## Abstract

Both iron and omega-3 (n-3) polyunsaturated fatty acids may play an important role in bone development. The aim of this study was to investigate the effects of pre- and post-natal iron and n-3 fatty acid deficiency (FAD), alone and in combination, on bone development in rats, and to determine whether effects are reversible when a sufficient diet is provided post-weaning. Using a 2×2-factorial design, 56 female Wistar rats were allocated to one of four diets: (1) control, (2) iron deficient (ID), (3) n-3 FAD or (4) ID and n-3 FAD, and were maintained on the respective diets throughout gestation and lactation. At weaning (post-natal day [PND] 21), offspring (*n* = 24/group; male:female=1:1) were randomly allocated to either continue with their respective diets or to switch to the control diet until PND 42-45. Bone mineral density (BMD) and bone strength were determined using dual X-ray absorptiometry and three-point bending tests, respectively. Pre- and post-natal ID resulted in significantly lower BMD in the spine and bone strength in the left femur. Both ID and n-3 FAD resulted in lower BMD in the right femur, with an additive reduction in the combined ID and n-3 FAD group vs. controls. While negative effects of pre- and post-natal ID alone were reversed in offspring switched to a control diet post-weaning, lower BMD and bone strength persisted in offspring with combined ID and n-3 FAD during the prenatal and early post-natal period. Effects were not sex-specific. These results indicate that ID during early life may negatively influence bone development, with potential additive effects of n-3 FAD. While the effects of ID alone seem reversible, a combined ID and n-3 FAD may result in irreversible deficits in bone development.

## Introduction

Worldwide, osteoporosis causes ~9 million fractures annually (1). Bone tissue is constantly remodeled through resorption via osteoclasts and bone formation via osteoblasts. From infancy to young adulthood, bone increases in thickness and density until peak bone mass (PBM) is reached (2). Thereafter, bone loss will slowly start occurring, whereby bone resorption predominates over formation, and will continue until the end of life. It is widely recommended to promote the achievement of a high PBM during childhood or adolescence with the aim of decreasing the risk of osteoporosis later in life (3–7).

It has been suggested that 20–40% of the variance in PBM and osteoporosis risk is attributed to environmental factors, such as nutrition (3, 6). Several human studies have indicated that different maternal nutritional factors, such as calcium and fat intake as well as vitamin D status, can influence bone development in the offspring (8–11). Therefore, one of the International Osteoporosis Foundation Compendium's key priorities for 2020–2025 in the prevention of osteoporosis includes the promotion of optimal nutrition during pregnancy and childhood (7).

Iron intake may be another nutritional factor that can play a role in bone development (12, 13). Iron deficiency (ID) is the most prevalent nutrient deficiency globally (14). Pregnant women have an added risk due to iron stores often being insufficient to cover for the increased demands during pregnancy (15, 16). In elderly adults, serum iron, serum ferritin (iron-storage protein) and dietary iron intake have been positively associated with bone mineral density (BMD) (17–19). Additionally, ID anemia has been identified as a significant and independent risk factor for the development of osteoporosis in a Taiwanese population-based study (20). Several rodent studies have found that ID induced post-weaning can lower bone strength parameters (21–24).

Omega-3 (n-3) polyunsaturated fatty acids (PUFAs) are another group of nutrients that may influence bone development (25–28). Intakes of alpha-linolenic acid (ALA, 18:3n-3) and docosahexaenoic acid (DHA, 22:6n-3), both n-3 PUFAs, are often insufficient in pregnant and lactating women in developing countries (29). Several human studies have found positive effects of n-3 PUFA supplementation on bone health (30–34). However, rodent studies found conflicting results on the effects of maternal fatty acid (FA) intake on bone development in offspring (35–39).

Even though ID and n-3 fatty acid deficiency (FAD) may affect bone development via shared or independent mechanisms, data on potential interactions between ID and low n-3 FA status on bone development do exist, to our knowledge, not exist. Therefore, the aims of this study were: (1) to investigate the effects of pre- and post-natal iron and n-3 PUFA deficiency, alone and in combination, on bone development in rats, and (2) to determine whether effects are reversible when offspring are switched to a control diet post-weaning. We further explored whether effects are sex-specific.

## Materials and Methods

### Animals and Diets

All Wistar rats in this experiment were bred and housed in a temperature and humidity-controlled (22 ± 2°C and 55 ± 15% relative humidity) animal facility (Vivarium of the Preclinical Drug Development Platform of the North-West University [NWU] in Potchefstroom, South Africa) with a reversed 12-h dark-light cycle (lights off at 06:00 a.m.). The experiments have been conducted following the ARRIVE guidelines for animal research (40). Ethical approval has been obtained from the AnimCare Ethics Committee (National Health Research Ethics Council reg. number AREC-130913-015) of the Faculty of Health Sciences of the NWU (NWU-00566-19-A5).

The purified experimental diets were obtained commercially from Dyets Inc. (Bethlehem, USA) and were based on the American Institute of Nutrition (AIN) 93G purified diets for laboratory rodents for growth, pregnancy and lactation (41). All diets were isocaloric and contained 10% fat. The basal AIN-93G formulation (control diet) contained 40 mg/kg iron, soybean oil at 70 g/kg diet and hydrogenated coconut oil at 30 g/kg (42–44). The ID diets were modified to contain 15–18 mg/kg diet iron (45), while the FAD diets contained 81 g/kg diet hydrogenated coconut oil and 19 g/kg diet safflower oil (46–48). Ingredients of the diets are presented in Supplementary Table 1 and the analyzed fatty acid and iron composition of the diets is presented in Supplementary Table 2.

### Experimental Design

**Figure 1:**
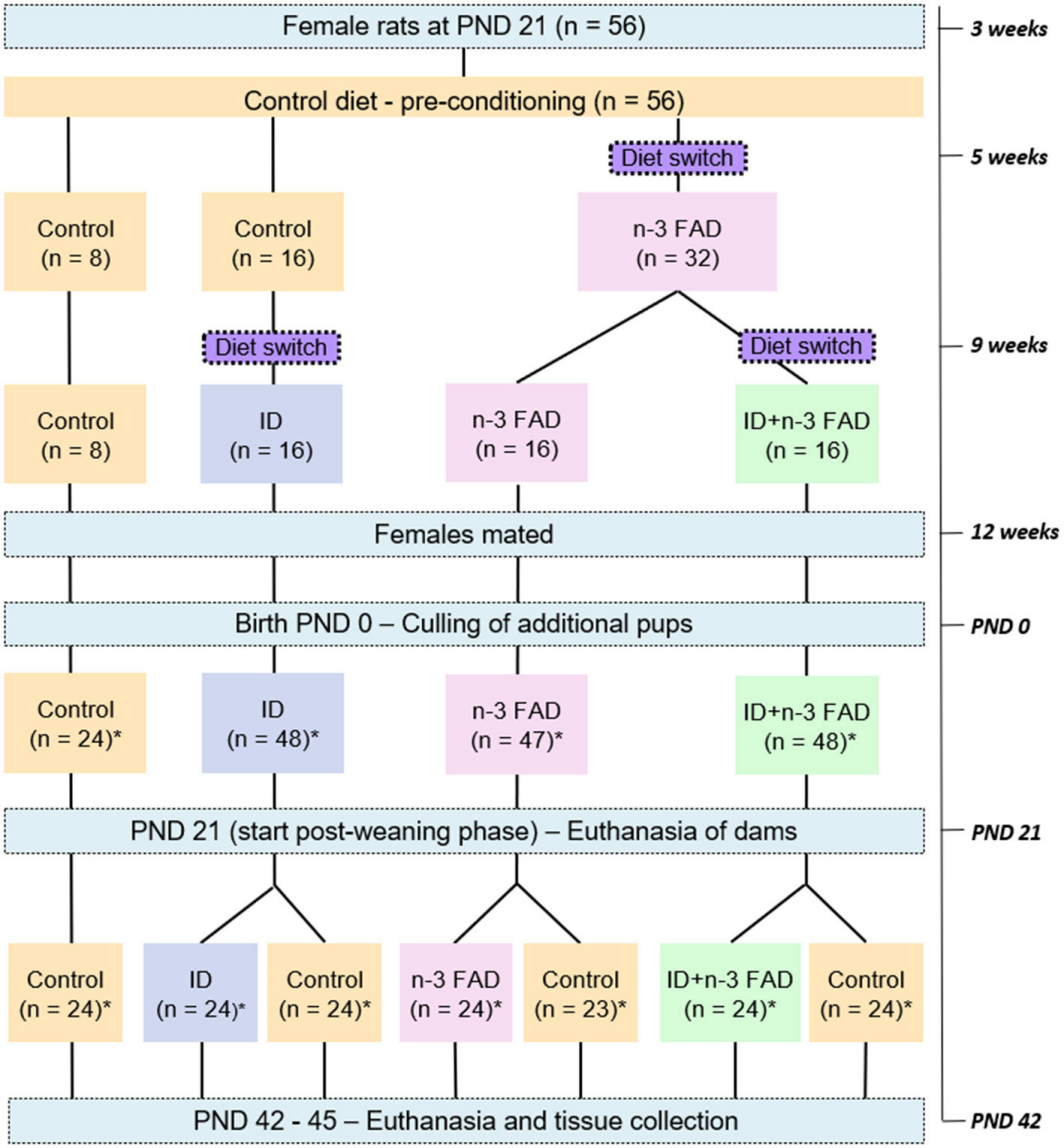
Schematic diagram of the experimental design. n, number of rats; PND, post-natal day; ID, iron deficiency; n-3 FAD, omega-3 fatty acid deficiency; *equal male:female ratio.

Figure 1 provides a schematic diagram of the experimental design. A total of 56 female Wistar rats (21 ± 3 days of age; minimum weight of 25 g) were pair-housed in standard individually ventilated solid floor cages (Techniplast GR900 SealSafe® Plus; 300 mm × 300 mm × 189 mm) with low trace element (< 2.00 ppm iron) alpha-cellulose bedding (Alpha-Dri® LBS Serving Biotechnology Ltd, Horley, UK). All rats had *ad libitum* access to purified diets and demineralized water (18 M*Ω*) and received a control diet for 2 weeks of preconditioning. At 5 weeks of age, the rats were randomly allocated (using a computerized random number generator—Excel) to one of four diet groups: (1) Control; (2) ID; (3) n-3 FAD; or (4) ID+n-3 FAD. Rats that were allocated to one of the n-3 FAD diet groups (n-3 FAD and ID+n-3 FAD) were placed on an n-3 FAD diet for seven weeks before mating, in order to imitate a chronic n-3 FAD diet (49). At 9 weeks of age, the rats that were allocated to one of the ID diet groups (ID or ID+n-3 FAD) were placed on the ID or ID+n-3 FAD diet, respectively, for 3 weeks before mating (50). At 12 weeks of age, female rats were mated with 12-week-old males, and maintained on their pre-gestation diets throughout gestation and lactation.

Three to 5 days after birth, the litters were culled to eight pups, ideally four males and four females per litter, to standardize nutritional adequacy between litters. Body weight was measured three times per week per litter. The remaining pups were weaned from the dams at post-natal day (PND) 21. Pups (*n* = 24/group; male:female=1:1; one too few female pup was born for the FAD-Control group) were then randomly allocated to either maintain their respective experimental diets (ID/ID, n-3 FAD/n-3 FAD and ID+n-3 FAD/ID+n-3 FAD) or to receive the control diet (ID/Control, n-3 FAD/Control and ID+n-3 FAD/Control) for 3 weeks. Body weight was measured three times per week individually from PND 21 until PND 42-45, when the offspring (*n* = 167) were euthanized by decapitation and tissue samples collected.

Sample sizes were determined by power calculations appropriate for animal model studies in nutrition research as reported by Ricci et al. (51). Researchers were blinded for diet groups when conducting weight measurements, sample collections and analyses.

### Sample Collection, Storage, and Analyses

After decapitation, trunk blood was collected in 4 ml ethylenediaminetetraacetic acid (EDTA)-coated vacutainer tubes (BD, Plymouth, United Kingdom) and centrifuged within 1 h at 3000 x g for 10 min at 4°C to separate plasma from buffy coat and red blood cells (RBCs). Red blood cells were washed twice with 0.15 mol NaCl/L by centrifugation at 3000 x g for 10 min. Plasma and RBC aliquots were stored at −80°C until analysis.

Hemoglobin concentrations were measured in fresh whole blood using a portable HemoCue® Hb 201+ photometer (HemoCue AB, Angelholm, Sweden).

Liver tissue was removed immediately after decapitation, snap-frozen in liquid nitrogen and stored at −80°C until analysis. Liver samples were homogenized and digested with nitric acid according to Erikson et al. (52), and total iron concentrations were measured by using the hydrogen reaction mode on an Agilent 7900 quadrupole ICP-MS at the Central Analytical Facilities, Stellenbosch University, South Africa. Samples were introduced via a 0.4 ml/min micromist nebulizer into a peltier-cooled spray chamber at a temperature of 2°C. The instrument was optimized for analysis in high matrix introduction mode, and all samples and standards were diluted with argon gas to minimize matrix load to the analyzer. The instrument was calibrated using a National Institute of Standards and Technology (NIST) traceable standard (Inorganic Ventures, USA). NIST-traceable quality control standards at high and low concentration levels (De Bruyn Spectroscopic Solutions, Bryanston, South Africa) were analyzed to verify the accuracy of the calibration before sample analysis commenced and this was repeated for every 12 samples to monitor drift. A germanium internal standard was introduced online to monitor instrument drift and correct for matrix differences between samples and standards. During the course of the analysis, internal standard recovery was between 90 and 110% for all samples, and recovery for drift monitor standards between 95 and 105%. Oxide formation were <0.3%. Three replicate measurements were completed for each sample.

Total phospholipid FA analysis in RBCs (% total FA) was performed using a modification of the method by Folch et al. (53). Briefly, total phospholipid FA fractions were isolated by TLC, trans-methylated to yield FAME, and analyzed by quadrupole GC-EI-MS on an Agilent Technologies 7890 A GC system equipped with an Agilent Technologies 5975C VL mass selective detector as previously described (42).

The lumbar spines and right femurs were collected with surrounding tissue, placed into 50 ml and 15 ml Falcon tubes, respectively, with phosphate-buffered saline and stored at −20°C until analysis. Analysis of BMD (g/cm2) was done *ex vivo* using dual X-ray absorptiometry (DXA) (Hologic Discovery W DXA scanner with APEX system software version 2.3.1).

The left femurs were collected and cleaned of any surrounding tissue, placed into 15 ml Falcon tubes with phosphate-buffered saline (PBS) and stored at −20°C until analysis. Three-point bending tests were performed at the mid-diaphysis of the left femur, at room temperature, using a servo-hydraulic machine (Instron model 1026). Bones were consistently oriented for testing in the anterior-posterior direction. The bottom supports (3 mm wide with rounded points) were placed 8 mm apart, with a perpendicular force (load) exerting pressure at the midpoint. Mechanical properties, such as ultimate load (a complete break, measured in newton [N]), ultimate displacement (deflection at the point of ultimate load, measured in mm), ultimate stress (ultimate load over cross-sectional area, measured in megapascal [MPa; N/mm2]) were determined from force deformation curves. During elastic deformation no damage is incurred on the bone, therefore the bone will return to its original state and internal strength if the load is removed during this stage. The bone stiffness was determined by the gradient of the linear line (elastic region) of the load over deformation, where a higher stiffness is evident of a more brittle bone. At the point of transition from the elastic to the plastic region (after which the bone is damaged permanently due to structural changes but not necessarily completely broken yet), the load, deformation and stress were also determined.

Insulin-like growth factor 1 (IGF-1) was analyzed in plasma using rat IGF-1 enzyme-linked immunosorbent assay (ELISA) kits (Sandwich-ELISA principle) provided by Elabscience (Wuhan, China) following the manufacturer's instructions.

Body weight, hemoglobin, liver iron, FA markers and BMD were measured in all pups within the different diet groups. Some measurements, however, were unsuccessful and the total usable numbers are reported in section Results. Plasma ferritin and IGF-1 were measured only in a random sub-sample of 12 pups per group. A random sample of left femurs were also used to optimize the three-point bending tests and were therefore also not used in statistical analyses.

### Statistical Analyses

Statistical analyses were performed using IBM SPSS Statistics software (version 25) and Excel 2016. Data were examined for normality of distribution (using q-q plots, histograms, and Shapiro-Wilk test) and the presence of outliers (using box plots). Homogeneity of variance were examined by the Levene's test. Variables that deviated significantly from normality and/or variance of homogeneity were transformed prior to interferential statistical analysis using analysis of covariance (ANCOVA).

The effects of pre- and post-natal ID and n-3 FAD, as well as their interactions, on BMD and indices of bone strength at PND 42-45 were determined using 2 x 2 ANCOVA (iron [sufficient vs. deficient] x n-3 FA [sufficient vs. deficient]). Significant main effects of ID and n-3 FAD in the absence of a significant interaction effect indicate additive effects of the deficiencies, whereas a significant interaction implies synergism or antagonism.

We further determined whether there was a sex [male vs. female] x diet interaction. When no significant interaction with sex was obtained, sex was included in the models as a covariate. As body weight can influence bone development (54) and ID and/or n-3 FAD may influence body weight, it was also included as a covariate. In the presence of a significant main effect or interaction, between-group differences were determined using one-way ANCOVA with sex and body weight as covariates, followed by Bonferroni's *post-hoc* test. Cross-sectional area of the femur was also included as a covariate for the outcome variables femur load and displacement, as the size of a bone can influence the strength thereof (55). No adjustment for cross-sectional area was made for ultimate or transition stress, however, as the area is used to calculate the stress.

Data are expressed as means ± standard error of means (SEM) (parametric data) or geometric means ± SEM (non-parametric data), and the significance of all tests was set at *P* <0.05.

## Results

### Growth, Iron, and n-3 Fatty Acid Status

Table 1 shows indices of body weight, iron and n-3 FA status at PND 42-45 for offspring that remained on their respective experimental diets and for those that were switched to a control diet post-weaning.

**Table 1:** Offspring body weight, iron, and n and omega-3 fatty acid status at post-natal day 42–45.

	**Geometric means ±SEM**								***P*** **-value**		
	**Control**	**n**	**ID/ID**	**n**	**n-3 FAD/n-3 FAD**	**n**	**ID+n-3 FAD/ID+n-3 FAD**	**n**	**ID**	**n-3 FAD**	**ID x n-3 FAD**
Body weight (g)	190 ± 1.0^a^	24	172 ± 1.0^ab^	24	188 ± 1.0^a^	24	157 ± 1.0^b^	24	<0.001	0.051	0.105
Weight gain from PND 21 (g)	133 ± 1.1^a^	24	114 ± 1.0^bc^	24	125 ± 1.0^ab^	24	105 ± 1.0^c^	24	<0.001	0.009	0.724
Hemoglobin (g/dL)	13.4 ± 1.0^a^	24	9.3 ± 1.0^b^	24	12.7 ± 1.0^a^	24	8.5 ± 1.0^b^	23	<0.001	0.044	0.582
Liver iron (μg/L)	142 ± 1.1^a^	24	27 ± 1.0^b^	24	169 ± 1.2^a^	22	26 ± 1.1^b^	24	<0.001	0.436	0.250
Plasma ferritin (ng/mL)	138 ± 1.3	11	121 ± 1.1	11	192 ± 1.5	11	193 ± 1.2	12	0.817	0.119	0.839
RBC n-3 FA (% total FA)	7.7 ± 1.0^a^	24	7.6 ± 1.0^a^	23	1.7 ± 1.0^b^	24	1.8 ± 1.1^b^	24	0.398	<0.001	0.213
RBC n-6 FA (% total FA)	42.6 ± 1.0^c^	24	42.2 ± 1.0^c^	23	48.9 ± 1.0^a^	24	47.0 ± 1.0^b^	24	0.003	<0.001	0.071
RBC n-6/n-3 FA (% total FA)	6.3 ± 1.0^b^	24	6.4 ± 1.0^b^	23	79.2 ± 1.1^a^	24	65.3 ± 1.1^a^	24	0.264	<0.001	0.223
	**Control**		**ID/Control**		**n-3 FAD/Control**		**ID+n-3 FAD/Control**				
Body weight (g)	190 ± 1.0^a^	24	184 ± 1.0^ab^	24	196 ± 1.0^a^	23	169 ± 1.0^b^	24	<0.001	0.377	0.014
Weight gain from PND 21 (g)	133 ± 1.1^a^	24	127 ± 1.0^ab^	24	127 ± 1.0^ab^	23	114 ± 1.1^b^	24	0.007	0.020	0.300
Hemoglobin (g/dL)	13.4 ± 1.0	24	13.7 ± 1.0	24	13.7 ± 1.0	22	13.7 ± 1.0	22	0.610	0.819	0.766
Liver iron (μg/L)	142 ± 1.1	24	140 ± 1.2	24	142 ± 1.2	23	94 ± 1.1	21	0.090	0.134	0.031
Plasma ferritin (ng/mL)	138 ± 1.3	11	193 ± 1.4	11	217 ± 1.3	12	282 ± 1.4	11	0.218	0.123	0.859
RBC n-3 FA (% total FA)	7.7 ± 1.0^a^	24	7.9 ± 1.1^a^	21	6.0 ± 1.0^b^	22	5.9 ± 1.0^b^	20	0.987	<0.001	0.659
RBC n-6 FA (% total FA)	42.6 ± 1.0^bc^	24	41.7 ± 1.0^c^	20	44.9 ± 1.0^a^	22	43.6 ± 1.0^b^	22	<0.001	<0.001	0.582
RBC n-6/n-3 FA (% total FA)	6.3 ± 1.0^b^	24	5.6 ± 1.0^b^	19	9.0 ± 1.0^a^	22	8.9 ± 1.0^a^	20	0.097	<0.001	0.154

*Two-way ANCOVA was used to test effects of ID, n-3 FAD, and ID x n-3 FAD interactions, adjusted for sex. All parameters were log transformed to perform ANCOVA. Between-group differences were determined using one-way ANCOVA followed by Bonferroni's post-hoc test (adjusted for sex). Values are unadjusted geometric means ± SEM for log transformed variables. Means in a row with superscripts without a common letter differ (P < 0.05)*.

*ID, iron deficiency; n-3 FAD, omega-3 fatty acid deficiency; FA, fatty acids; n-6 FA, omega-6 fatty acids; PND, post-natal day; RBC, red blood cell*.

Both pre- and post-natal ID and n-3 FAD resulted in lower body weight at PND 42-45 (ID, *p* < 0.001; n-3 FAD, *p* = 0.051) and lower body weight gain from PND 21 (ID, *p* < 0.001; n-3 FAD, *p* = 0.009) in offspring that remained on their respective diets until PND 42-45. Only the ID+n-3 FAD groups had significantly lower body weight compared to the Control group, while both ID groups (ID/ID and ID+n-3FAD/ID+n-3FAD) gained significantly less weight from PND 21 compared to the Control group.

In offspring switched to a control diet post-weaning, there was an ID x n-3 FAD interaction on body weight at PND 42-45 (*p* = 0.014); only the ID+n-3 FAD/Control offspring persisted with lower body weight compared to the Control group. The effect of ID and n-3 FAD for lower body weight gain from PND 21 persisted in offspring switched to a control diet post-weaning (ID, *p* = 0.007; n-3 FAD, *p* = 0.020), with only the ID+n-3 FAD/Control group having significantly lower weight gain compared to the Control group.

In offspring that remained on their respective diets, there were effects of both ID and n-3 FAD for lower hemoglobin concentrations at PND 42-45 (ID, *p* < 0.001; n-3 FAD, *p* = 0.044). However, only the ID groups (ID and ID+n-3 FAD) had significantly lower hemoglobin concentrations than the Control group. A pre- and post-natal ID further resulted in lower liver iron stores (ID, *p* < 0.001) in both ID groups, while plasma ferritin concentrations were not affected by ID or n-3 FAD.

In offspring that were switched to a control diet post-weaning, the effects of ID and n-3 FAD on hemoglobin did not persist. There was, however, an ID x n-3 FAD interaction on liver iron (*p* = 0.031); only the ID+n-3 FAD/Control offspring persisted having lower liver iron concentrations compared to the Control group. No significant effects were observed on plasma ferritin concentrations.

Pre- and post-natal n-3 FAD resulted in a lower percentage of n-3 FA (n-3 FAD, *p* < 0.001), a higher percentage of n-6 FA (n-3 FAD, *p* < 0.001) and a higher n-6/n-3 FA ratio (n-3 FAD, *p* = 0.001) in RBC total phospholipids of offspring that remained on their respective diets. Furthermore, there was an effect of ID for lower RBC n-6 FA (ID, *p* < 0.003), resulting in lower RBC n-6 FA in the ID+n-3 FAD/ID+n-3 FAD group than the n-3 FAD/n-3 FAD group.

In offspring switched to a control diet post-weaning, the significant effects of n-3 FAD for a lower percentage of n-3 FA, a higher percentage of n-6 FA and a higher n-6/n-3 ratio persisted, even though levels were higher when compared to their non-switched counterparts. Also, the effect of ID for lower RBC n-6 FA did persist in the offspring switched to a control diet.

### Spine and Right Femur Bone Mineral Density

**Figure 2:**
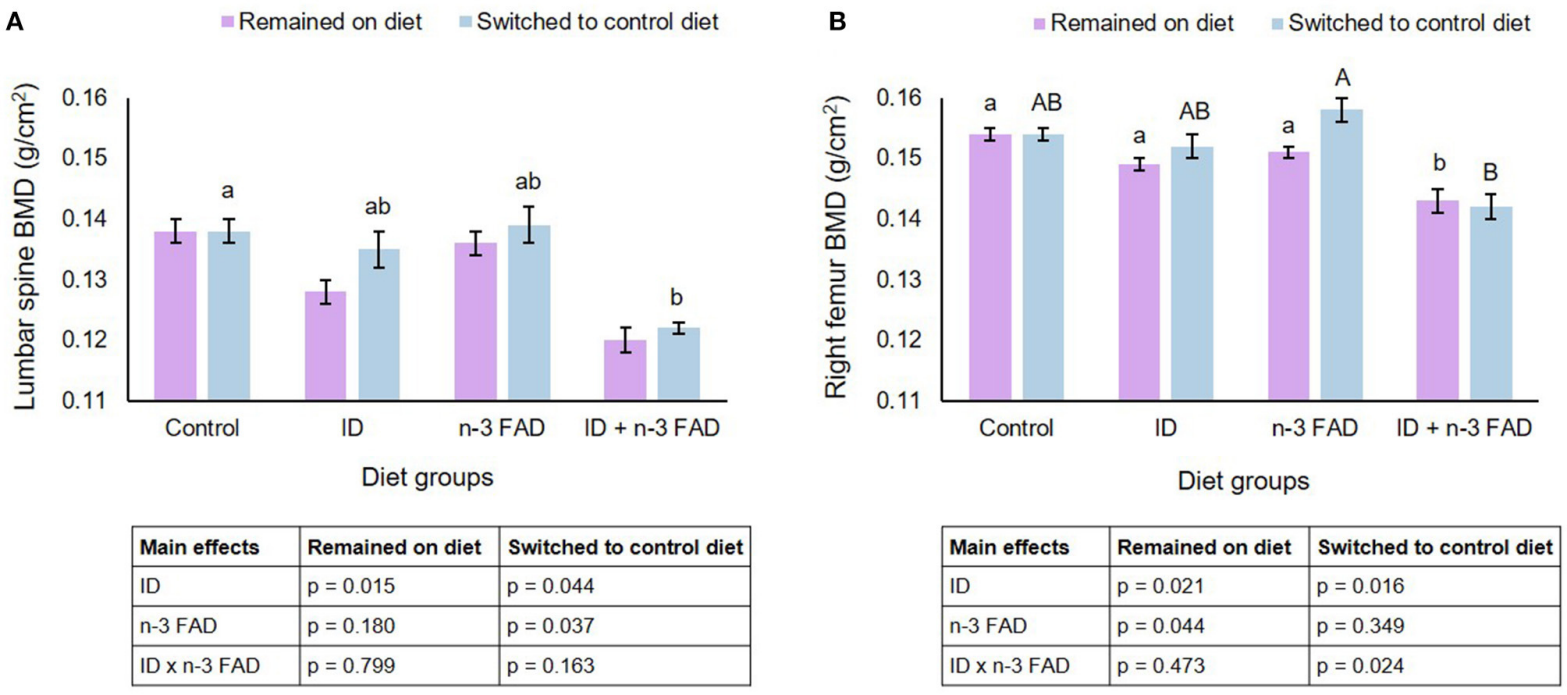
Lumbar spine and right femur bone mineral density. Two-way ANCOVA was used to test effects of ID, n-3 FAD, and ID x n-3 FAD interactions, adjusted for sex and body weight. Spine and right femur BMD were log transformed to perform ANCOVA. Between-group differences were determined using one-way ANCOVA followed by Bonferroni's *post-hoc* test (adjusted for sex and body weight). Values are unadjusted geometric means ± SEM for log transformed variables. Means with superscripts without a common (capital or small) letter differ (*P* < 0.05). ID, iron deficiency; n-3 FAD, omega-3 fatty acid deficiency; BMD, bone mineral density.

Pre- and post-natal ID resulted in lower BMD in the lumbar spine (Figure 2A) (ID, *p* = 0.015) of offspring that remained on their respective diets until PND 42-45. Both ID and n-3 FAD resulted in lower BMD in the right femur (Figure 2B) (ID, *p* = 0.021; n-3 FAD, *p* = 0.044), with the ID+n-3 FAD/ID+n-3 FAD group having significantly lower BMD in the right femur compared to the Control group.

In offspring that were switched to a control diet post-weaning, both ID and n-3 FAD resulted in lower BMD in the spine (ID, *p* = 0.044; n-3 FAD, *p* = 0.037), and the ID+n-3 FAD group had significantly lower BMD than the Control group. There was an ID x n-3 FAD interaction for right femur BMD (*p* = 0.024); a lower BMD persisted only in the previously ID+n-3 FAD offspring when compared to the n-3 FAD/Control group.

No diet x sex interactions were observed on BMD in the spine and right femur.

### Left Femur Size and Bone Strength Indicators

Table 2 shows left femur size and bone strength indicators at PND 42-45 for offspring that remained on the respective experimental diets and for those that were switched to a control diet post-weaning.

**Table 2:** Left femur size and bone strength indicators in offspring at post-natal day 42–45.

	**Means** **±SEM for different groups**				***P*** **-value**		
	**Control**	**ID/ID**	**n-3 FAD/n-3 FAD**	**ID+n-3 FAD/ID+n-3 FAD**	**ID**	**n-3 FAD**	**ID x n-3 FAD**
Femur weight (mg)	609 ± 17^ab^	569 ± 23^ab^	614 ± 14^a^	515 ± 17^b^	0.604	0.628	0.001
Femur length (mm)	17.9 ± 1.01	17.8 ± 1.02	17.7 ± 1.01	17.0 ± 1.01	0.752	0.057	0.069
Femur area (mm^2^)	4.3 ± 0.1	4.1 ± 0.1	4.0 ± 0.1	3.8 ± 0.1	0.036	0.505	0.745
Stiffness (N/mm)	114.7 ± 8^ab^	80.2 ± 7^ab^	110.5 ± 6^a^	72.2 ± 6^b^	0.003	0.554	0.716
Ultimate load (N)	43.2 ± 1.7^a^	32.4 ± 1.6^b^	42.4 ± 1.2^a^	31.2 ± 1.2^b^	<0.001	0.884	0.659
Transition load (N)	31.4 ± 1.6	22.5 ± 1.4	30.9 ± 1.4	21.9 ± 1.5	0.011	0.754	0.720
Ultimate displacement (mm)	0.76 ± 0.04	0.80 ± 0.03	0.86 ± 0.04	0.86 ± 0.06	0.089	0.011	0.549
Transition displacement (mm)	0.30 ± 0.03	0.32 ± 0.04	0.30 ± 0.02	0.33 ± 0.03	0.158	0.607	0.966
Ultimate stress (MPa)	10.0 ± 1.1^a^	7.9 ± 1.0^b^	10.5 ± 1.0^a^	8.2 ± 1.0^b^	<0.001	0.559	0.812
Transition stress (MPa)	7.3 ± 0.35^ab^	5.6 ± 0.42^b^	7.8 ± 0.47^a^	5.8 ± 0.39^b^	0.004	0.492	0.574
	**Control**	**ID/Control**	**n-3 FAD/Control**	**ID+n-3 FAD/Control**			
Femur weight (mg)	609 ± 17^ab^	633 ± 23^a^	620 ± 13^a^	528 ± 19^b^	0.692	0.108	<0.001
Femur length (mm)	17.9 ± 0.1^a^	17.8 ± 0.2^ab^	17.7 ± 0.1^ab^	17.2 ± 0.1^b^	0.047	0.017	0.237
Femur area (mm^2^)	4.3 ± 0.1	4.1 ± 0.1	4.0 ± 0.1	3.7 ± 0.1	0.853	0.165	0.120
Stiffness (N/mm)	114.7 ± 8^a^	111.6 ± 12^a^	111.5 ± 7^a^	62.3 ± 8^b^	0.003	0.001	0.003
Ultimate load (N)	43.2 ± 1.7^a^	40.0 ± 1.3^a^	41.3 ± 1.4^a^	30.7 ± 1.9^b^	0.003	0.007	0.057
Transition load (N)	31.4 ± 1.6	30.0 ± 2.0	28.8 ± 1.7	21.7 ± 1.4	0.321	0.053	0.382
Ultimate displacement (mm)	0.76 ± 0.04	0.71 ± 0.04	0.86 ± 0.04	0.85 ± 0.04	0.775	0.003	0.491
Transition displacement (mm)	0.30 ± 0.03^b^	0.34 ± 0.05^ab^	0.28 ± 0.02^b^	0.40 ± 0.05^a^	0.005	0.138	0.082
Ultimate stress (MPa)	10.0 ± 1.1^a^	9.8 ± 1.0^a^	10.3 ± 1.0^a^	8.0 ± 1.1^b^	0.006	0.031	0.005
Transition stress (MPa)	7.3 ± 0.35	7.4 ± 0.45	7.2 ± 0.41	5.9 ± 0.46	0.354	0.138	0.158

*Two-way ANCOVA was used to test effects of ID, n-3 FAD and ID x n-3 FAD interactions, adjusted for sex and body weight. Femur length and ultimate stress were log transformed to perform ANCOVA. Between-group differences were determined using one-way ANCOVA followed by Bonferroni's post-hoc test (adjusted for sex and body weight). Means in a row with superscripts without a common letter differ (P < 0.05). Values are unadjusted means ± SEM or geometric means ± SEM for log transformed variables. (Control, n = 15; ID/ID, n = 12; n-3 FAD/n-3 FAD, n = 19; ID+n-3 FAD/ID+n-3 FAD, n = 13; ID/Control, n = 14; n-3 FAD/Control, n = 20; ID+n-3 FAD, n = 12). ID, iron deficiency; n-3 FAD, omega-3 fatty acid deficiency; BMD, bone mineral density*.

There was an ID x n-3 FAD interaction for femur weight (*p* = 0.001) in offspring that remained on their respective diet until PND 42-45; Femur weight was significantly lower in ID+n-3FAD/ID+n-3 FAD group compared to the n-3 FAD/n-3 FAD group. There were no significant effects observed on femur length when adjusting for both sex and body weight. However, both pre- and post-natal ID and n-3 FAD (ID, *p* = 0.022; n-3 FAD, *p* = 0.007) lowered femur length when adjusting for sex only. Pre- and post-natal ID resulted in a smaller mid-diaphysis cross-sectional femur area (ID, *p* = 0.036) but no significant differences were found between diet groups.

In offspring switched to a control diet post-weaning, there was an ID x n-3 FAD interaction on femur weight (*p* < 0.001); femur weight was significantly lower in the previously ID+n-3 FAD (ID+n-3 FAD/Control) offspring when compared to the ID/Control and n-3 FAD/Control offspring. When adjusting for sex only, femur weight persisted to be significantly lower in the previously ID+n-3 FAD offspring compared to the Control group. Femur length was affected by both ID and n-3 FAD (ID, *p* = 0.047; n-3 FAD, *p* = 0.017), with the previously ID+n-3 FAD group having significantly lower femur length than the Control group. In the switched offspring, ID and n-3 FAD no longer had effects on femur area when adjusting for sex and body weight, however, both ID and n-3 FAD resulted in smaller femur area when adjusting for sex only (ID, *p* = 0.038; n-3 FAD, *p* = 0.009).

In offspring that remained on their respective diets until PND 42-45, ID significantly reduced femur stiffness, ultimate load, transition load, ultimate stress and transition stress, with both ID groups (ID and ID+n-3 FAD) having significantly (or tending to have significantly) lower values compared to the Control group. Effects of ID on stiffness (ID, *p* = 0.003), ultimate load (ID, *p* = 0.001), and transition load (ID, *p* = 0.032) persisted even after adjusting for bone area, in addition to sex and body weight. There were no effects of n-3 FAD on these bone strength indices. However, n-3 FAD did increase ultimate displacement (*p* = 0.011).

Switching offspring to a control diet post-weaning resulted in ID x n-3 FAD interactions on femur stiffness (*p* = 0.003) and ultimate stress; lower femur stiffness and ultimate stress persisted only in the previously ID+n-3 FAD offspring (ID+n-3 FAD/Control) when compared to the Control group. An effect of ID (*p* = 0.003) and n-3 FAD (*p* = 0.007) persisted for ultimate load, resulting in the previously ID+n-3 FAD offspring having significantly lower ultimate load compared to the Control group. Furthermore, ID pre-weaning increased transition displacement (*p* = 0.005), but only resulted in a significantly higher transition replacement in the previously ID+n-3 FAD group when compared to the Control group.

No diet x sex interactions were observed on any of the femur size or bone strength indices.

### Insulin-Like Growth Factor-1

**Figure 3:**
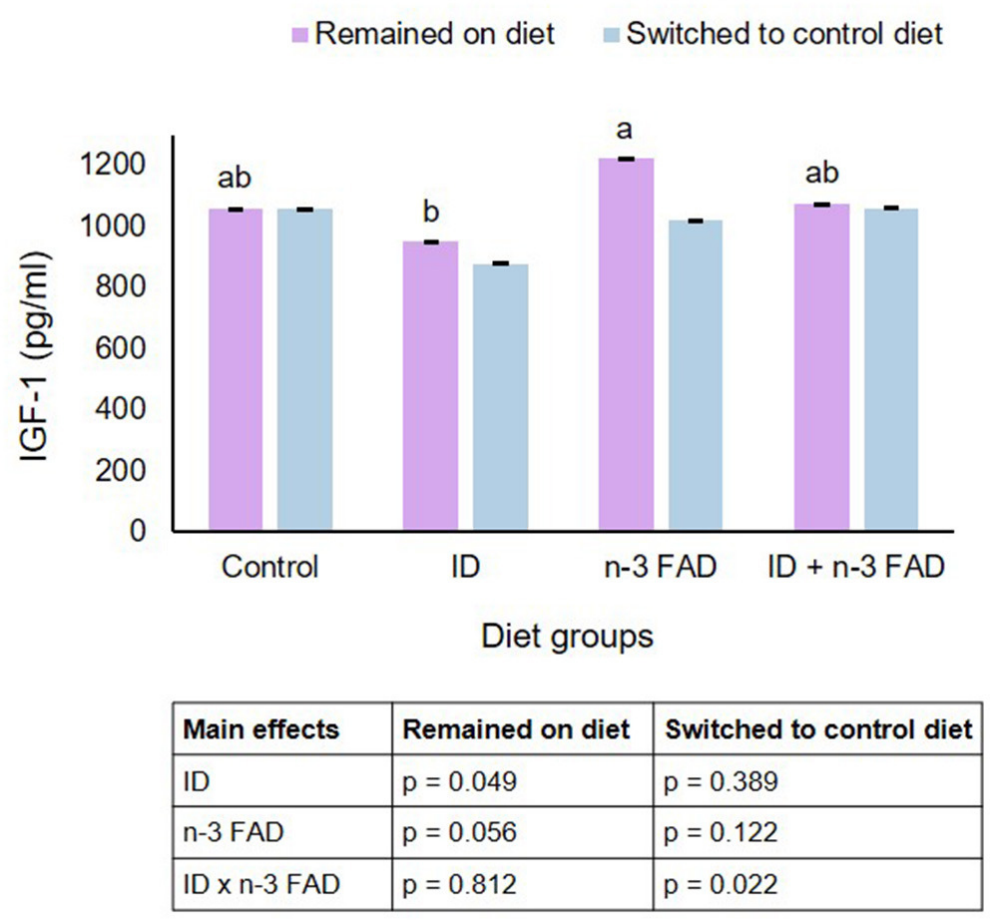
Insulin-like growth factor-1. Two-way ANCOVA was used to test effects of ID, n-3 FAD, and ID x n-3 FAD interactions, adjusted for sex and body weight. IGF-1 was log transformed to perform ANCOVA. Between-group differences were determined using one-way ANCOVA followed by Bonferroni's *post-hoc* test (adjusted for sex and body weight). Means with superscripts without a common letter differ (*P* < 0.05). ID, iron deficiency; n-3 FAD, omega-3 fatty acid deficiency; IGF-1, insulin-like growth factor-1.

In offspring that remained on their respective diets post-weaning, ID significantly lowered IGF-1 (ID, *p* = 0.049), while n-3 FAD tended to elevate IGF-1 (n-3 FAD, *p* = 0.056) (Figure 3). In offspring switched to a control diet post-weaning, there was a significant ID x n-3 FAD interaction on IGF-1 (*p* = 0.022), and the ID/Control group tended to differ from the ID+FAD/Control group (p = 0.055). There were no diet x sex interactions observed on IGF-1. Furthermore, there were no correlations between IGF-1 concentrations and BMD or any bone strength parameters in offspring that remained on their respective diets or offspring that were switched to a control diet.

## Discussion

To the authors' knowledge, this is the first study to investigate the effects of pre- and post-natal ID and n-3 FAD, alone and in combination, on bone development, as well as the reversibility of effects when switching offspring to a sufficient diet post-weaning. The results from this study show that ID throughout gestation, lactation and up to PND 42-45 (adolescence/early adulthood) lowers BMD in the spine and bone strength in the femur, and that combined ID and n-3 FAD additively lowers BMD in the femur. This study further indicates that switching offspring to the control diet from PND 21 can reverse the negative effects of ID alone on BMD and bone strength, while ID results in irreversible deficits in bone development when combined with n-3 FAD. Observed effects were not sex-specific.

More specifically, the results from the three-point-bending tests showed that pre- and post-natal ID leads to bones that are more prone to deformation (lower stiffness; less brittle) and that withstand less forces up to the point of damage (transition load) and the total breaking point (ultimate load). Even though, to the authors' knowledge, no previous studies determined the effect of ID during pregnancy on bone development in the offspring, studies have shown that ID can lower BMD and bone strength parameters when rats are fed an ID diet post-natally (21, 22, 24). A recent review has proposed that the effect of ID on bone may be dependent on ID severity (56). The iron content of the experimental diets in previous rodent studies varied between 5 and 8 mg iron/kg diet (21, 22) or was not specified (24). In this study, diets contained 15–18 mg iron/kg, highlighting that even a moderate ID during early development can lead to detrimental effects on bone development.

Little is known about the exact mechanisms by which ID affects bone development, but iron has been suggested to act as a cofactor of enzymes involved in collagen bone matrix synthesis and to play a role in the conversion of the biologically inactive form of vitamin D to its active form 1,25-dihydroxyvitamin D (12). Studies in both Gambian women (57) and mice (58) have shown that maternal ID can lead to elevated serum concentrations of fibroblast growth factor-23 [FGF-23] in infants. FGF-23 is a bone-derived hormone suppressing 1,25-dihydroxyvitamin D synthesis in the kidney and phosphate absorption, which could lead to hypo-mineralization and consequently softening and weakening of bones (59). However, the effect of maternal ID on direct measures of bone development and health, such as BMD, in the offspring remains uncertain. Furthermore, ID has been suggested to affect the differentiation of osteoblasts, possibly through the bone morphogenetic pathway (56).

In the current study, both ID and n-3 FAD resulted in lower BMD in the right femur, with a significant additive reduction in the combined ID and n-3 FAD groups when compared to the Control group. Conflicting results have been found on the effects of maternal FA status on bone development in the offspring, with some animal studies showing a positive relationship between n-3 FA status and bone outcomes (36–39) and others showing no relationship (35). Inconsistent results may be attributed to different dietary n-3 FA concentrations and composition, different time periods of exposure, different strains of animals used, and different outcomes and time points of outcomes measured. It has been proposed that perinatal n-3 FAD may influence bone development by decreasing calcium absorption or by influencing osteoblastogenesis and osteoclastogenesis via various complex pathways (25–28, 60).

Lower BMD and bone strength observed in offspring exposed to pre- and post-natal ID alone were reversed in offspring switched to an iron-sufficient diet post-weaning. There are no previous studies investigating whether the negative effects of ID on bone outcomes are reversible, but a recent trial in Kenyan women showed that iron supplementation during pregnancy can reverse elevated FGF-23 production caused by ID in the ID mothers and their infants (61). In contrast, lower BMD and bone strength persisted in offspring that developed on a combined ID and n-3 FAD diet during the prenatal and early post-natal period. Therefore, our results highlight that a combined ID and n-3 FAD during early life elicits more severe and potentially irreversible effects on bone development than an ID or n-3 FAD alone. The mechanisms underlying the observed additive and irreversible effects of ID and n-3 FAD on bone development are unclear. However, the additive nature of the effects of ID and n-3 FAD on the measured bone outcomes indicates that ID and n-3 FAD affect these outcomes via independent mechanisms. It is likely that the impact of the combined deficiency on bone metabolism is severe enough to lead to irreversible deficits in bone development.

There are demonstrated sex-based differences in bone structure development early in life (62). While males and females acquire bone mass at similar rates before puberty, males achieve a greater bone mass than females after puberty and show differences in bone size and geometry, suggesting sex hormones to be the primary determinants of sex-related differences in pubertal bone growth (62). Thus, we hypothesized that the impact of ID and n-3 FAD on outcomes related to bone development, particularly during adolescence, could be sex-dependent. However, results from this study did not support this hypothesis. In contrast, previous studies found that the effects of n-3 PUFA supplementation on various neonatal bone outcomes are sex-dependent (37, 38). The authors speculated that the observed differences in the effects of maternal supplementation on male and female offspring might be due to divergent sex-specific regulation of long-chain PUFA metabolism (38).

IGF-1 is a major hormone promoting bone growth, and studies in animals and humans have shown that nutritional deficiencies can affect IGF-1 signaling (63). In the current study, pre- and post-natal ID resulted in lower plasma IGF-1, while n-3 FAD tended to result in higher IGF-1 concentrations. Consequently IGF-1 concentrations in the n-3 FAD group were higher than in the ID group. However, IGF-1 concentrations did not correlate with BMD or any bone strength parameters in this study.

Strengths of this study include the 2×2 factorial design investigating the effects of ID and n-3 FAD, alone and combined, on bone development. Most nutrient deficiencies seldom occur in isolation and it is likely that women of child-bearing age may suffer from both ID and n-3 FAD (64, 65). Another strength of this study is the amount of iron provided in the ID diets to induce an ID severity in the dams and offspring similar to what can be expected in a human context. Similarities in the pathophysiologic responses between the human and rat skeleton make the rat a valuable model for research on bone health (66). Studies in humans and computer simulations have shown that achieving a higher PBM early in life will decrease the risk of osteoporosis later in life (3, 5, 67). The tracking of bone strength indicators from childhood to adolescence further supports the notion that prevention of osteoporosis should begin in the early stages of bone development (68–70). Increasing bone density or bone strength early in life may also have current benefits to children or adolescents, e.g., in reducing their fracture risk (3).

The main limitation of this study was the inability to follow offspring up to late adulthood because it formed part of a larger study. Thus, we were not able to determine whether effects remain and affect fracture risk later in life. Furthermore, we did not determine food intake, and did not include pair-fed control groups to control for the effects reductions in food intake caused by the deficiencies might have had on the measured outcome variables. However, potential differences in food intake were at least partly controlled for by adding body weight as a covariate in the statistical models. It is also important to acknowledge that while lower Hb and liver iron concentrations were reversed, lower n-3 FAs and an elevated n-6/n-3 FA ratio in RBCs persisted after switching offspring to a control diet for 3 weeks post-weaning. Therefore, the observed persisting effects of ID in combination with n-3FAD might have differed with a longer exposure to an n-3 PUFA-sufficient diet post-weaning.

In conclusion, this study found that ID during early life may negatively influence bone development, with potential additive effects when ID is combined with n-3 FAD. While the effects of ID alone seem reversible, a combined ID and n-3 FAD may result in irreversible deficits in bone development. Additional research is needed to confirm whether these effects are long-lasting, and whether effects can be reversed with maternal iron and/or n-3 FA supplementation during gestation and lactation.

## Data Availability Statement

The raw data supporting the conclusions of this article will be made available by the authors, without undue reservation.

## Ethics Statement

The animal study was reviewed and approved by the AnimCare Ethics Committee (National Health Research Ethics Council reg. number AREC-130913-015) of the Faculty of Health Sciences of the NWU (NWU-00566-19-A5).

## Author Contributions

ES, LZ, CS, HK, and JB conceptualized and designed the study. ES, LZ, EK, and JB executed the study and collected data. ES, LZ, PV, and JB performed biochemical analyses. ES and JB performed the statistical analyses and wrote the first draft of the manuscript, and have primary responsibility for its final content. All authors reviewed and approved the final manuscript.

## Funding

This work was supported by the National Research Foundation of South Africa (grant number 92748) and Inqaba Biotec's SeedIT Initiative. The funders had no involvement in the study design, collection, analysis, and interpretation of data, or the writing of this paper.

## Author Disclaimer

Any opinion, finding and conclusion or recommendation expressed in this material is that of the author(s) and the NRF does not accept any liability in this regard.

## Conflict of Interest

The authors declare that the research was conducted in the absence of any commercial or financial relationships that could be construed as a potential conflict of interest.

## Publisher's Note

All claims expressed in this article are solely those of the authors and do not necessarily represent those of their affiliated organizations, or those of the publisher, the editors and the reviewers. Any product that may be evaluated in this article, or claim that may be made by its manufacturer, is not guaranteed or endorsed by the publisher.
