# Supplementary material for: Effects and Reversibility of Pre- and Post-natal Iron and Omega-3 Fatty Acid Deficiency, Alone and in Combination, on Bone Development in Rats
Source: Front Nutr. 2022 Jan 17;8:802609. doi: 10.3389/fnut.2021.802609 (PMC8801778; doi:10.3389/fnut.2021.802609)
Supplement: Supplementary file 1 [file Table_1.DOCX]

Supplementary Material

# Supplementary Tables

Supplemental Table 1: Ingredients of experimental diets based on the AIN-93G diet

|  | **Control** | **ID** | **n-3 FAD** | **ID+n-3 FAD** |
| --- | --- | --- | --- | --- |
| Cornstarch (g/kg) | 397.5 | 397.5 | 397.5 | 397.5 |
| Casein (g/kg) | 200 | 200 | 200 | 200 |
| Dyetrose – Dextrinised cornstarch (g/kg) | 132 | 132 | 132 | 132 |
| Sucrose (g/kg) | 100 | 100 | 100 | 100 |
| Soybean oil (g/kg) | 70 | 70 | - | - |
| Hydrogenated coconut oil (g/kg) | 30 | 30 | 81 | 81 |
| Safflower oil (g/kg) | - | - | 19 | 19 |
| Vitamin mix (g/kg) | 10 | 10 | 10 | 10 |
| Mineral mix (g/kg) | 35 | 35^1^ | 35 | 35^1^ |

*^1^Mineral mix was modified in the ID diets to contain 15 – 18 mg iron/kg diet*

Supplemental Table 2: Analyzed iron and fatty acid composition of diets

|  | **Control** | **ID** | **n-3 FAD** | **ID+n-3 FAD** |
| --- | --- | --- | --- | --- |
| Iron (ppm) | 41.3 | 15.2 | 43.4 | 15.6 |
| Total Fatty Acids (g/100g) | 9.78 | 9.78 | 9.76 | 9.76 |
| Saturated Fatty Acids (g/100g) | 3.82 | 3.82 | 7.53 | 7.53 |
| Monounsaturated Fatty Acids (g/100g) | 1.45 | 1.45 | 0.33 | 0.33 |
| Polyunsaturated Fatty Acids (g/100g) | 3.95 | 3.95 | 1.32 | 1.32 |
| Trans Fatty Acids (g/100g) | 0.083 | 0.083 | 0.039 | 0.039 |
| Omega 3 Fatty Acids (g/100g) | 0.495 | 0.495 | 0.009 | 0.009 |
| Omega 6 Fatty Acids (g/100g) | 3.64 | 3.64 | 1.37 | 1.37 |
| Omega 9 Fatty Acids (g/100g) | 1.41 | 1.41 | 0.327 | 0.327 |
| 20:3 Eicosatrienoic (g/100g) | <0.007 | <0.007 | <0.007 | <0.007 |
| 20:4 Arachidonic (g/100g) | <0.007 | <0.007 | <0.007 | <0.007 |
| 20:5 Eicosapentaenoic (g/100g) | <0.007 | <0.007 | <0.007 | <0.007 |
| 22:5 Docosapentaenoic (g/100g) | <0.007 | <0.007 | <0.007 | <0.007 |
| 22:6 Docosahexaenoic (g/100g) | <0.007 | <0.007 | <0.007 | <0.007 |
